# Evaluation of Hsp70 and Apoptotic Markers in Canine Cutaneous Lupus Erythematosus

**DOI:** 10.3390/vetsci13040369

**Published:** 2026-04-11

**Authors:** Gian Enrico Magi, Gabiria La Gamba, Francesca Mariotti, Lucia Biagini, Giacomo Rossi, Alessandro Di Cerbo

**Affiliations:** School of Biosciences and Veterinary Medicine, University of Camerino, Via Circonvallazione 93/95, 62024 Matelica, Italy; gianenrico.magi@unicam.it (G.E.M.); gabiria.lagamba@studenti.unicam.it (G.L.G.); francesca.mariotti@unicam.it (F.M.); lucia.biagini@unicam.it (L.B.); giacomo.rossi@unicam.it (G.R.)

**Keywords:** Hsp70, apoptosis, dog, cutaneous lupus erythematous, autoimmune disease

## Abstract

Heat shock proteins 70 (HSP 70) are molecular chaperonins ubiquitously expressed in both prokaryotes and eukaryotes and are involved in the modulation and exacerbation of the immune response. The present study aimed to assess the immunohistochemical expression of HSP70 and apoptosis markers, such as TUNEL and Caspase-3, in 17 cases of cutaneous lupus erythematosus (CLE) in dogs to determine whether HSP70 expression correlates with cell apoptosis and to highlight possible involvement of HSP70 in the pathogenesis of CLE. The results revealed positive HSP70 expression in epidermal and inflammatory cells across all cases, with a significant correlation between HSP70 expression score and TUNEL-positive cells but not with Caspase-3-positive cells. This correlation could indicate a possible role for HSP70 in cell death via a caspase-independent apoptotic mechanism or other programmed cell death mechanisms, such as pyroptosis or necroptosis. The precise mechanisms by which HSP70 acts in this specific pathological context remain incompletely understood, but the results of this study provide important information for future investigations into autoimmune skin disease in dogs.

## Simple Summary

Heat shock proteins 70 (HSP 70) are involved in the modulation and exacerbation of the immune response in dogs affected by cutaneous lupus erythematosus. In our study, HSP 70 expression significantly correlated with TUNEL, an apoptotic marker. Our results, although preliminary, aim to fill a gap in the understanding of HSP70’s role in a specific pathological context, such as autoimmune diseases in dogs.

## 1. Introduction

Lupus erythematosus (LE) is an autoimmune disease affecting dogs and humans, with a multifactorial etiology that can be restricted to the skin (cutaneous lupus erythematosus—CLE) or systemic (systemic lupus erythematosus—SLE). In LE, the mechanisms that maintain B- and T-cell self-tolerance are impaired, resulting in the production of autoantibodies against nuclear and cytoplasmic components (e.g., histones, nonhistone proteins bound to RNA, double-stranded DNA, and nucleolar antigens). The tissue damage observed in LE is primarily mediated by a type III hypersensitivity reaction, in which immune complex deposition activates complement. This process subsequently recruits and activates neutrophils, triggering an inflammatory response, or promotes antibodies binding to target cells, leading to phagocytosis. To a lesser extent, type II hypersensitivity reactions, mediated by antibodies directed against self-antigens on various cell types, and type IV cell-mediated hypersensitivity reactions also contribute to LE pathogenesis [1,2].

The form of lupus most frequently diagnosed in dogs is CLE, with the variant called discoid lupus erythematosus (DLE) characterized by lesions typically confined to the face. German shepherds are predisposed to this disease. DLE primarily affects the *nasal planum* and dorsal muzzle and rarely involves the pinnae, lips, and periocular region. Clinically, it presents with lesions, such as erythematous macules, papules, or plaques, often accompanied by erosions, although these findings are not pathognomonic [3,4]. Histologically, CLE is characterized by a cell-rich, cytotoxic interface dermatitis with abundant lymphocytes, vacuolar degeneration and/or apoptosis of basal keratinocytes and mild thickening of the basal membrane [4,5]. However, similar histopathological features may also be observed in other conditions, such as leishmaniasis and immune-mediated dermatoses like erythema multiforme. Various endogenous and exogenous factors have been implicated in the etiology of LE. In DLE, facial lesions are exacerbated by ultraviolet light exposure; consequently, this form is also referred to as nasal solar dermatitis [3,4].

Heat shock proteins (HSPs) are stress-induced proteins that are upregulated during tissue damage and generally play a protective role. Their expression is triggered in cells exposed to sublethal heat shock [6,7]. HSPs are present in both eukaryotic and prokaryotic organisms and are essential for maintaining proteostasis and protecting cells from stressors [8,9]. In human medicine, altered expression of heat shock protein 70 (HSP 70) has been reported in several autoimmune diseases, including multiple sclerosis and SLE. In particular, abnormal HSP70 expression in skin lesions has been suggested to contribute to both lesion development and the production of SLE-associated antibodies [10,11]. In the pathogenesis of SLE, type 1 interferons play a key role, and their activity appears to be influenced by HSPs [12]. Type 1-interferons are produced by plasmacytoid dendritic cells (plasma cells that undergo morphological transformation post-activation), and their production can be enhanced by HSPs, particularly HSP70 [12]. These findings highlight an apparent paradox between the well-established protective functions of HSPs and the potential pathogenetic role of HSP70 in LE. Furthermore, the involvement of HSPs in apoptosis has been investigated in several human diseases, yielding controversial results [11].

The expression of HSP70 in canine CLE has not yet been investigated. Therefore, this study aims to address this gap by evaluating the expression of HSP70 and apoptosis markers, such as Caspase-3 and TUNEL, in skin biopsies from dogs with CLE in order to explore the potential role of HSP70 in the pathogenesis of this disease.

## 2. Materials and Methods

In this study, seventeen skin samples from the *planum nasale* and muzzle of dogs with CLE were considered. All dogs suffered from chronic dermatitis involving the *planum nasale* that, in 7 cases, extended to the dorsum of the muzzle. Grossly crusted, eroded, and ulcerated skin lesions were reported. In 5 cases, clinicians reported worsening of the lesions after exposure to sunlight. The samples were submitted between 2013 and 2025 to the Pathology Unit of the School of Biosciences and Veterinary Medicine of the University of Camerino (Italy) as punch biopsies. The samples were fixed in 10% neutral buffered formalin and embedded in paraffin. Subsequently, serial sections (3 μm) were cut and stained with hematoxylin and eosin (HE). The diagnosis was established histopathologically when the following microscopic findings were present: lymphohistiocytic interface dermatitis (predominantly lymphocytes with a band-like lichenoid appearance) of varying degrees (diffuse or multifocal), primarily with a subepidermal distribution and located around hair follicles; hydropic degeneration and apoptosis (Civatte bodies) of basal keratinocytes; and melanophages within the superficial dermis (pigmentary incontinence). The cases considered involved dogs of different breeds, aged between 2 and 10 years, with 10 males and 7 females. All cases were negative on the ELISA serological test for *Leishmania* (Leishcan, Esteve), negative for the anti-nuclear antibody test (ANA), and negative on the immunohistochemical analysis using a monoclonal anti-Kinetoplast Membrane Protein-11 (KPM-11) antibody for the presence of *Leishmania* organisms in skin samples. The clinical and anamnestic data for all cases are shown in Table 1.

### 2.1. Immunohistochemical Analysis and Immunoreactivity Scoring System for HSP70 and Caspase 3

The immunohistochemical analysis was performed using a standard ABC–peroxidase method on 3 μm thick sections, as described by Mariotti et al. [13]. All sections were deparaffinized and rehydrated using a Leica Autostainer XL (Milan, Italy) and then immersed in a 4% hydrogen peroxide solution in distilled water and incubated in the dark at room temperature (RT) for 1 h to inhibit endogenous peroxidases. Subsequently, antigenic unmasking was performed by heating the sections in a microwave oven at 750 W for 10 min in citrate buffer (pH 6). After, the samples were allowed to cool to room temperature and then immersed in Tris-buffered saline (TBS). Sections were demarcated with a DakoPen, and 100 μL of normal goat serum (NGS) diluted 1:10 with a solution of 1% bovine serum albumin (BSA), and 1% polyvinylpyrrolidone (PVP) solubilized in TBS was added to each section, which was then incubated for 1 h at room temperature. Subsequently, a mouse monoclonal anti-HSP70 antibody (5A5, Abcam, Cambridge, UK) and a rabbit polyclonal anti-Caspase 3 antibody (CPP32, Thermo Scientific, Milan, Italy), both diluted 1:50 in the same solution as for NGS, were added to different serial sections and incubated overnight in a moist chamber at RT. After incubation, TBS washings were performed on the sections, and then goat anti-mouse and goat anti-rabbit biotinylated antibodies (Vector Laboratories, Newark, CA, USA) were added as secondary antibodies. ABC (Avidin–biotin complex) (Vectastain, Vector Laboratories) was added along with DAB chromogen (3,3-diaminobenzidine tetrahydrochloride). Sections were counterstained in Mayer’s hematoxylin. A Leica DM2500 light microscope (Leica DFC7000 T) was used to visualize reactions and capture images. For the negative control, a rabbit IgG-isotype control antibody (Abcam) and a mouse IgG1 isotype control antibody (Thermo Scientific, Milan, Italy) were used instead of the primary antibody. Each sample was examined at 10× and 20× magnification, with 5 microscopic fields evaluated in a semi-quantitative assessment of HSP70, as described by Magi et al. (2022), with modifications detailed in the subsequent text [14].

In more detail, each sample was scored based on the number of positive inflammatory cells, the number of positive epidermal cells, and signal intensity. Positive cell percentage was based on a 5-point scoring system: 0 = no positive cells; 1 = positive cells ranging from 0 to 25%; 2 = positive cells ranging from 26 to 50%; 3 = positive cells ranging from 51 to 75%; 4 = positive cells > 75%. For each microscopic field, the counts of immunopositive and immunonegative cells were recorded and then expressed as percentages. The intensity of immunostaining was scored using a 4-point scale: 0 = no staining; 1 = low intensity; 2 = moderate intensity; 3 = high intensity.

In samples with heterogeneous intensity, the chosen score was the predominant one. The overall score assigned to each case was derived by multiplying the cell positivity and intensity signal scores, with a minimum score of 0 and a maximum of 12. All cases were scored by three authors (G.E.M., G.R., and F.M.), and in cases of equivocality, reviewed by all authors involved in scoring to establish the score. For the caspase-3 evaluation, a semi-quantitative count of cells in the epidermis undergoing apoptosis was performed. For each sample, three microscopic fields were evaluated: a 40× objective, a 10× eyepiece, and a square eyepiece grating (10 × 10 squares, with a total area of 62,500 μm^2^).

### 2.2. TUNEL Assay

Samples were prepared for immunohistochemical analysis, but instead of adding the primary antibody, the sections were stained with the DeadEnd™ Colorimetric Terminal deoxynucleotidyl transferase dUTP nick end labeling (TUNEL) system (Thermo Scientific, Milan, Italy). Next, the secondary antibody (goat anti-mouse, 1:200) was applied, and the same steps as previously described for IHC were followed. A semiquantitative count of positive cells was performed as described above.

### 2.3. Statistical Analysis

Data were analyzed using GraphPad Prism 9 software (GraphPad Software, Inc., La Jolla, CA, USA). A nonparametric Spearman correlation test was used to assess the correlation between Caspase 3 and HSP70, and between TUNEL and HSP70, as continuous variables. A *p* < 0.05 was defined as statistically significant.

## 3. Results

**Figure 1:**
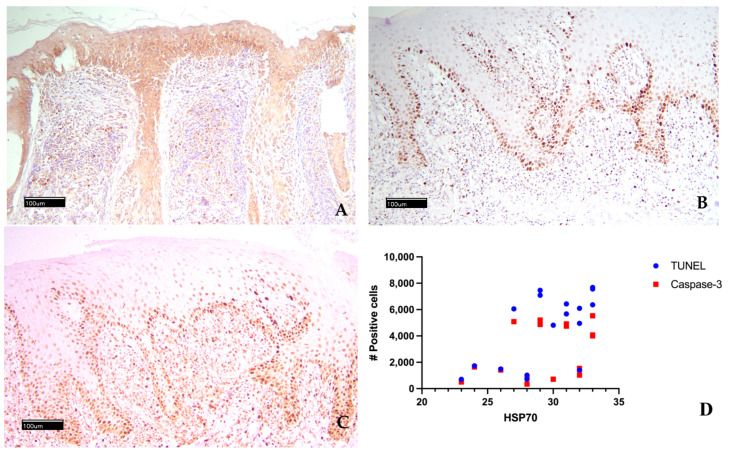
Epidermis diffusely positive for HSP70 with high-intensity immunostaining (**A**); numerous epidermal basal cells and suprabasal cells are TUNEL-positive (**B**); numerous epidermal basal cells and suprabasal cells are immunostained with Caspase 3 (**C**); non-parametric Spearman test correlation for TUNEL and HSP70 (**D**).

The HSP70 expression, TUNEL assay, and Caspase 3 expression results are shown in Table 2 and Figure 1. The scores assigned for HSP70 expression across the 17 cases by the three raters were identical, indicating total inter-observer agreement. In all 17 CLE cases studied, epidermal keratinocytes were immunopositive for HSP70, and in 14 cases, the basal and suprabasal layers were strongly immunostained. Nine cases also showed positivity for inflammatory cells in the superficial dermis, and in 11 cases, the immunosignal intensity was high. The immunopositivity of epithelial–epidermal cells was cytoplasmic; membrane reinforcement was rare. From the descriptive examination of the results of the assigned scores, the lowest score was 7.6 (case n. 4), while the highest score of 11 was assigned to three cases (cases n. 4, 9, 15), and in three other cases, a very high score of 10.7 was assigned (cases n. 11, 13, 17). The average score was 9.82 ± 1. For Caspase 3, the mean number of immunopositive cells was 2816 ± 1991; positivity ranged from a maximum of 5525 cells in case n. 5 to a minimum of 346 in case n. 7. Two other cases had more than 5000 positive cells (cases n. 3 and 12). For TUNEL, the mean number of immunopositive cells was 4543 ± 2691, with positivity ranging from a maximum of 7683 cells in case n. 15 to a minimum of 706 in case n.4. Three other cases had more than 7000 positive cells (cases n. 9, n. 12, n. 16). TUNEL- and Caspase 3-positive cells had nuclear staining.

Comparing the immunohistochemical HSP70 score with the TUNEL-positive cell count, eight of the nine cases with the highest HSP70 score (10 to 11) had a high number of TUNEL-positive cells (>4000). The same trends were not observed when comparing the HSP70 score with the cell immunopositivity for Caspase-3. The statistical analysis showed a significant correlation between TUNEL-positive cells and HSP70 score (*p* < 0.05), while the correlation between Caspase 3-positive cells and HSP70 score was not significant (*p* = 0.1938).

## 4. Discussion

Although some HSPs are constitutively produced by cells, most function as molecular chaperones in response to stimuli that cause protein denaturation [10,15]. These damaging stimuli include heat, nutritional deficiencies, oxidative stress, acute and chronic inflammation, viral and bacterial infections, ischemia, heavy metals, and excessive exercise. HSPs therefore play a cytoprotective role against protein denaturation, facilitating the repair of misfolded peptides and the degradation of irreparably damaged proteins. This function is linked to their involvement in protein folding and in the translocation of organelles across cellular membrane, which is why they have been defined as chaperonins [10,11,15]. These highly conserved proteins also participate in additional functions, including signal transduction and mitochondrial ROS generation, and they can induce apoptosis in cells [16]. In this regard, several studies have highlighted a potential pathogenic role of HSPs; indeed, HSP malfunction has been associated with numerous human diseases [11]. In the pathogenesis of many inflammatory diseases, excessive cellular apoptosis is implicated as a consequence of increased free radical levels, which amplify the inflammatory response [11,17]. Under physiological conditions, ROS levels and the inflammatory response are tightly regulated by cellular antioxidant mechanisms; however, under pathological conditions, this balance may be disrupted, leading to oxidative stress and extensive cell death [11]. Maintaining a balance between inflammatory and oxidant-antioxidant states is therefore essential. In this context, HSPs modulate inflammatory cascades that lead to endogenous ROS generation and intrinsic apoptosis by inhibiting proinflammatory factors, thereby playing a crucial role in the pathogenesis of inflammatory diseases and cancer.

The involvement of HSPs in apoptosis has been investigated in both animal and human studies. In most cases, HSPs suppress apoptotic pathways; however, HSP60 has been shown to exhibit pro-apoptotic activity. Complexes of HSP60 with HSP10 localized in mitochondria can activate pro-caspase 3 in cytochrome-c dependent apoptosis [18,19,20]. Other studies have demonstrated that exogenous HSP60 can induce apoptosis in certain diseases via a TLR4/NF-κB-caspase signaling pathway [18,21]. These observations suggest that the role of HSPs in apoptosis is complex and sometimes controversial [10,11,15]. Interestingly, the same stress signals that trigger apoptosis also stimulate the expression and release of HSPs.

The role of HSPs has been studied in numerous human disorders, including cancer, neurodegenerative diseases, cardiovascular diseases, and autoimmune diseases. Studies on immune response to HSP in inflammatory and autoimmune diseases have shown that can elicit both immunoregulatory and immunostimulatory effects, highlighting a bimodal and sometimes paradoxical role in autoimmunity [10].

In autoimmune diseases, small HSPs not only act as chaperonins but also play an important role in cytoskeleton organization in response to stress. Aberrant phosphorylation of HSP27 has been demonstrated in various autoimmune skin diseases, such as pemphigus vulgaris and pemphigus foliaceus, as well as in myasthenia gravis [22,23].

HSP40 generally exhibits proinflammatory activity in autoimmune diseases. Molecular mimicry between human HSP60 and non-human HSP65 can trigger autoimmune reactions, and autoantibodies against HSP60/65 have been detected in diseases such as systemic lupus erythematosus, Sjögren’s syndrome, rheumatoid arthritis, and autoimmune hepatitis [10]. Type 1 diabetes mellitus is an autoimmune disease, and experimental models have demonstrated that HSP60 can induce both a T-cell immune and an anti-HSP60 antibody response that worsens disease progression. At the same time, HSPs have also been shown to stimulate a Th2 response, thereby inhibiting the progression of diabetes [24]. Immunization with mycobacterial HSP65 has been shown to prevent disease progression in non-obese diabetic mice by inducing a Th2 response with increased IL-10 and IL-4 production and associated downregulation of the Th1 response [25]. HSP70 has been shown to induce immunostimulatory responses in autoimmune diseases by activating antigen-presenting cells that secrete inflammatory cytokines (IL-12, TNFα, and IL-1β) [26]. In a study using the RIP-GP/P14 mouse model, immunization with recombinant HSP70 induced diabetes, suggesting a proinflammatory role in autoimmunity [26]. Moreover, HSP70/Ro52 and HSP70/Ro53 complexes promote increased infiltration of macrophages and cytotoxic T cells [27]. Elevated levels of autoantibodies against HSP70 have been observed in individuals with thyroiditis and diabetic microangiopathy, suggesting a role in immune modulation [10]. In a mouse model of salt-sensitive hypertension, circulating anti-HSP70 antibodies have been correlated with increased renal inflammation [28].

Studies on rheumatoid arthritis have highlighted HSP70 overexpression in fibroblast-like synoviocytes (RA-FLs), with modulatory effects on T-lymphocyte-mediated immune responses [10,11]. In vitro suppression of HSP70 via siRNA reduces inflammation by protecting RA-FLSs from nitric oxide-mediated apoptosis [29]. This finding suggests a potentially pro-apoptotic and detrimental role of HSP70 in rheumatoid arthritis, indicating that its inhibition could represent a therapeutic strategy. Similarly, in asthma, a chronic multifactorial inflammatory disease, HSP70 overexpression of HSP70 has been observed and correlates with disease severity [10,11]. Although the precise mechanism of HSP70 activity in asthma remains unclear, evidence suggests that it targets T lymphocytes and antibody-mediated immunity in response to infectious agents, potentially linking infection-induced immune responses with autoimmune processes [30]. Further studies have shown upregulation of HSP90 and HSP72 during ROS-induced asthma attacks in children, suggesting that elevated HSP levels may help refold denatured proteins following oxidative stress, thereby preventing protein aggregation, cellular damage, and further complications [31]. Increased HSP70 levels have been detected in peripheral blood mononuclear cells of patients with asthma, supporting their involvement in disease pathogenesis [32]. In systemic lupus erythematosus, HSP70 appears to play a role in the transport of autoantigens to the dermal–epidermal junction, where immune complexes are typically deposited. Its presence with these deposits in biopsy samples suggests involvement in the transfer of autoantigens from keratinocytes to the dermo-epidermal junction, thereby contributing to immune complex formation and inflammation [33]. Overall, the relationship between HSPs and autoimmunity in human medicine is complex, and some authors propose that HSP-based vaccination strategies may offer protective effects against autoimmune diseases [10,11].

In veterinary medicine, studies on HSPs have primarily focused on their role in stress responses and their expression in various tumor types. Increased HSP expression is generally considered an indicator of stress and is associated with their protective and anti-apoptotic function. Indeed, in livestock, dietary additives that enhance HSP expression have been shown to alleviate stress [15]. In veterinary oncology, several studies have demonstrated increased expression of HSPs in tumor cells, suggesting their potential use as diagnostic and prognostic biomarkers, as well as possible therapeutic targets [34,35].

To date, no studies have investigated the role of HSPs in the pathogenesis of autoimmune diseases in veterinary medicine. In the present study, we evaluated HSP70 expression in canine LE, a relatively common autoimmune skin disease, and assessed its correlation with cellular apoptosis using the TUNEL assay and caspase-3 expression. The results demonstrated high HSP70 expression in epidermal and inflammatory cells, with a significant correlation with TUNEL-positive epidermal cells but not with caspase-3-positive cells. An important finding was the positive correlation between elevated HSP70 expression and previously reported high TLR4 expression in the same cases [36]. Since HSP70 is an endogenous ligand of TRL4, their concurrent overexpression is plausible. Activation of TLR4 by HSPs in CLE, followed by the production of numerous pro-inflammatory cytokines, may exacerbate this chronic inflammatory condition through a positive feedback mechanism. HSP70 has also been shown to induce pro-inflammatory cytokine production via the MyD88/NFκB signaling pathway, involving TLR2 and TLR4, through CD14 [37]. The number of TUNEL-positive cells observed in the CLE cases exceeded the number of caspase-3-positive keratinocytes. The TUNEL assay detects DNA fragmentation, enabling identification of cells undergoing apoptosis, as well as other forms of DNA damage [38]. The positive correlation between HSP and TUNEL results may indicate a role for HSP70 in caspase-independent cell death mechanisms, either directly through a cytotoxic T cell-mediated response or indirectly via ROS-mediated modulation of apoptosis involving mitochondrial pathways. Given the dual role of HSP70 reported in the literature, other forms of programmed cell death, such as necroptosis and pyroptosis, may also be involved. These hypotheses warrant further investigation. Although preliminary, these findings contribute to a better understanding of the role of HSP70 in canine autoimmune diseases and provide a foundation for future research.

## Figures and Tables

**Table 1:** Essential clinical information of the 17 cases of CLE collected during the study (N = negative).

Case N°	Sex	Age	Breed	*Leishmania* Elisa-Test	ANA-Test	KPM-11 IHC Test
1	Male	5	Crossbreed	N	N	N
2	Female	5	German shepherd	N	N	N
3	Male	10	Cocker	N	N	N
4	Male	2	Maremma shepherd	N	N	N
5	Female	4	Collie	N	N	N
6	Male	3	Collie	N	N	N
7	Female	4	Crossbreed	N	N	N
8	Female	7	Crossbreed	N	N	N
9	Female	2	American Akita	N	N	N
10	Male	3	German shepherd	N	N	N
11	Male	2	Labrador	N	N	N
12	Male	5	Pinscher	N	N	N
13	Male	5	German shepherd	N	N	N
14	Female	6	Maremma shepherd	N	N	N
15	Male	4	Golden retriever	N	N	N
16	Male	6	Crossbreed	N	N	N
17	Female	8	Crossbreed	N	N	N

**Table 2:** Results of the final expression score for HSP70 and the count of TUNEL-positive and Caspase 3-positive cells in the studied cases.

Case N°	HSP70 Final Score	TUNEL	Caspase-3
1	9.30	1023	818
2	10.30	5668	4743
3	9.00	6053	5088
4	7.60	706	508
5	11.00	6359	5525
6	8.00	1730	1645
7	9.30	718	346
8	10.00	4809	713
9	11.00	7566	4074
10	8.70	1490	1409
11	10.70	1402	1028
12	9.70	7090	5201
13	10.70	4952	1457
14	10.30	6431	4912
15	11.00	7683	4008
16	9.70	7461	4870
17	10.70	6094	1535
Average	9.82	4543	2816
S.D.	1	2691	1991

## Data Availability

The original contributions presented in this study are included in the article. Further inquiries can be directed to the corresponding author.

## Abbreviations

The following abbreviations are used in this manuscript:

LE	Lupus erythematosus
CLE	Cutaneous lupus erythematosus
SLE	Systemic lupus erythematosus
DLE	Discoid lupus erythematosus
HSPs	Heat shock proteins
HE	hematoxylin and eosin
ANA	Anti-nuclear antibody test
KPM-11	Anti-Kinetoplast Membrane Protein-11
TBS	Tris-buffered saline
NGS	Normal goat serum
BSA	Bovine serum albumin
PVP	Polyvinyl-pyrrolidone
